# ILC2s expanded by exogenous IL-33 regulate CD45+CD11b+F4/80high macrophage polarization to alleviate hepatic ischemia-reperfusion injury

**DOI:** 10.3389/fimmu.2022.869365

**Published:** 2022-07-29

**Authors:** Hai-Ming Zhang, Xiao-Jie Chen, Shi-Peng Li, Jin-Ming Zhang, Jie Sun, Liu-Xin Zhou, Guang-Peng Zhou, Bin Cui, Li-Ying Sun, Zhi-Jun Zhu

**Affiliations:** ^1^ Liver Transplantation Center, National Clinical Research Center for Digestive Diseases, Beijing Friendship Hospital, Capital Medical University, Beijing, China; ^2^ Clinical Research Center for Pediatric Liver Transplantation of Capital Medical University, Beijing, China; ^3^ Department of Critical Liver Disease, Liver Research Center, Beijing Friendship Hospital, Capital Medical University, Beijing, China

**Keywords:** ischemia-reperfusion injury (I/R), group 2 innate lymphoid cells (ILC2s), Kupffer cells (KCs), M2 polarization, IL-4

## Abstract

Hepatic ischemia-reperfusion injury (IRI) is an adverse consequence of hepatectomy or liver transplantation. Recently, immune mechanisms involved in hepatic IRI have attracted increased attention of investigators working in this area. In specific, group 2 innate lymphoid cells (ILC2s), have been strongly implicated in mediating type 2 inflammation. However, their immune mechanisms as involved with hepatic IRI remain unclear. Here, we reported that the population of ILC2s is increased with the development of hepatic IRI as shown in a mouse model in initial stage. Moreover, M2 type CD45+CD11b+F4/80high macrophages increased and reached maximal levels at 24 h followed by a significant elevation in IL-4 levels. We injected exogenous IL-33 into the tail vein of mice as a mean to stimulate ILC2s production. This stimulation of ILC2s resulted in a protective effect upon hepatic IRI along with an increase in M2 type CD45+CD11b+F4/80high macrophages. In contrast, depletion of ILC2s as achieved with use of an anti-CD90.2 antibody substantially abolished this protective effect of exogenous IL-33 and M2 type CD45+CD11b+F4/80high macrophage polarization in hepatic IRI. Therefore, this exogenous IL-33 induced potentiation of ILC2s appears to regulate the polarization of CD45+CD11b+F4/80high macrophages to alleviate IRI. Such findings provide the foundation for the development of new targets and strategies in the treatment of hepatic IRI.

## Introduction

Impairments in liver function resulting from ischemia-reperfusion injury (IRI) in liver transplantation or partial hepatectomy represent a significant adverse factor that not only affects the recovery, but may also contribute to the high morbidity and mortality rates associated with this procedure. With IRI, there exist numbers of possible sources of damage including that from oxidative stress, apoptosis, autophagy and pyroptosis, all of which can play important roles in occurrence and development of these adverse effects (1, 2). In addition, activities of immune cells can also affect hepatic IRI process. For example, tissue-resident macrophages in liver, play essential roles in hepatic IRI. As based on differences in their phenotypes and functions, activated macrophages can be divided into either M1 or M2 groups. During the initial stages of IRI, M1 macrophages produce reactive oxygen species (ROS) and proinflammatory factors (TNF-α, IL-1, IL-6) which can instigate this liver damage (3, 4). In contrast, in the later stages of IRI, polarized M2 macrophages secrete anti-inflammatory factors (Arg-1, IL-10, TGF-β, HO-1) to alleviate hepatic IRI (5, 6). However, these polarization mechanisms of M2 macrophages in hepatic IRI remain unclear.

Results from recent studies have shown that phenotypic changes in macrophages may be affected by group 2 innate lymphoid cells (ILC2s) (7). ILC2s, which are mainly found in lung, intestine, skin and liver, are tissue-resident cells derived from lymphoid progenitors (8). They can be activated by alarmins such as IL25, IL-33 after tissue damage (9, 10) and play crucial roles in metabolic homeostasis, parasite infection and tissue repair through inducing type 2 inflammation (11). In asthma, the number of ILC2s have been shown to be positively correlated with the number of M2 macrophages in induced sputum from asthmatic patients (12), and the expression of M2 macrophage related genes can be induced in co-cultures of ILC2s and Alveolar Macrophages (13). ILC2s can be found in various locations and exert a number of different roles depending on their current surroundings. For example, in bone marrow, ILC2s potentiate an IL-33 down-regulation of RANKL expression and transform bone marrow-derived monocytes/macrophages into M2 macrophage like cells through the production of granulocyte macrophage colony stimulating factor (GM-CSF) and IL-13 (14). As shown in studies related to inflammatory injuries, ILC2s also play a key role in tumor immunity and ILC2s proliferation can promote the pathogenesis of cancer by inducing M2 Macrophage polarization (15–17). Moreover, results from a recent report have indicated that ILC2s are involved in the repair process of damaged organs (18). Despite all this evidence regarding the immune mechanisms of ILC2s, such mechanisms as related to liver IRI have yet to be established.

Exogenous IL-33 is a potent stimulator of ILC2 proliferation (19), and a role for IL-33 in mouse models of hepatic IRI has been previously described. Li Shu et al. demonstrated that pretreatment with exogenous IL-33 reduced warm hepatic IRI in mice, and that this protective effect of IL-33 on hepatic IRI was mainly due to a Th1 to Th2 type shift (20). However, it remains unclear as to which group of cells are mainly involved in this effect. Further evidence for a protective effect of exogenous IL-33 on hepatic IRI was provided by Sakai et al (21). In contrast to these demonstrations of IL-33-induced protection, Yazdani reported that exogenous IL-33 exacerbated hepatic IRI by amplifying the neutrophil extracellular trap formation (22), and while Barbier (23) also reported a deleterious effect of endogenous IL-33 on liver IRI. Accordingly, this effect of IL-33 on hepatic IRI clearly warrants further study.

Work within our laboratory has indicated that levels of intrahepatic ILC2s vary as a function of the stage of IRI. Here, we reported that in the initial stages of IRI, as the time of reperfusion injury gradually progresses, ILC2s demonstrate a continuous upward trend, reaching peak levels at 12 h post-reperfusion. Interestingly, during this IRI period, there is a positive correlation between these changes in ILC2s and the proportion of M2 type CD45+CD11b+F4/80high macrophages. With a potentiation of ILC2s, as can be achieved with an exogenous administration of IL-33, intrahepatic IL-4 expression is also significantly increased followed by an increase in the proportion of M2 CD45+CD11b+F4/80high macrophages. Hepatocyte injury is dramatically mitigated in response to this IL-33 administration, while ILC2s depletion, as achieved with use of the anti-CD90.2 antibody, substantially abolished this protective effect of exogenous IL-33 and M2 CD45+CD11b+F4/80high macrophage polarization in hepatic IRI. When collating these findings, we found that ILC2s expanded by exogenous IL-33 alleviated hepatic IRI by promoting the M2 polarization of CD45+CD11b+F4/80high macrophages. Such findings provide the impetus for the development of new treatment strategies involving use of exogenous IL-33 and ILC2s in the treatment of IRI.

## Results

### Intrahepatic ILC2s vary as a function of hepatic IRI stage

**Figure 1:**
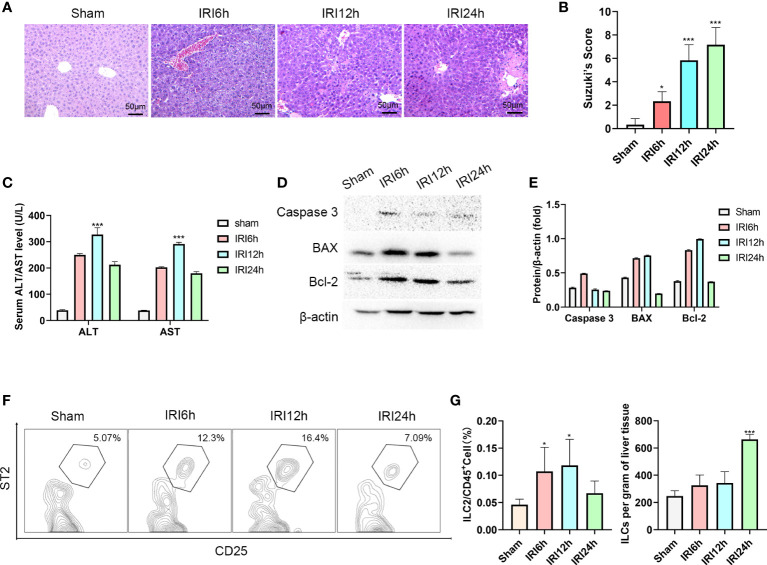
Intrahepatic ILC2s vary as a function of hepatic IRI stage. **(A)** HE staining of formalin fixed paraffin embedded liver tissue after ischemia followed by reperfusion at 6, 12 or 24 h and Sham controls (×200). **(B)** Suzuki’s scores for IR-induced liver injury among the four groups (n = 6 per group). **(C)** Serum ALT and AST levels were assessed in Sham and IRI groups at different post-reperfusion time periods. **(D, E)** western blot and semi-quantification of BAX, Bcl-2 and Caspase3 from liver tissue of Sham and IRI groups at different post-reperfusion time periods. **(F)** Proportion of ILC2s within the different IRI Groups. **(G)** Histograms of percent of ILC2s in CD45+cells and ILC2s per gram of liver tissue (n = 5 per group). (ns *P*>0.05, *P<0.05,***P<0.001 versus sham).

As an approach to determine whether liver-resident ILC2s change as a function of IRI stage, we established a mouse hepatic IRI model. Compared with that observed in the sham group, the main manifestations of hepatic IRI included hepatocyte edema, hepatic vessel congestion and necrosis. Mild edema and congestion were the main lesions present at 6 h post-IRI. At 12 h post-IRI, there was a progression of this damage with the gradual presence of punctate necrosis and inflammatory cell infiltration, while at 24 h, flake coagulation necrosis can be seen, but edema and congestion were reduced (**Figure 1A**). Quantified assessment of this hepatic IRI with use of Suzuki’s Scores demonstrated that the liver injury induced by ischemia-reperfusion was clearly time-dependent, with scores showing a gradual increase as a function of reperfusion time, reaching maximal levels at 24 h post-reperfusion (**Figure 1B**, *P*<0.001 versus sham). Results of Western blotting of Caspase-3, BAX and Bcl-2 in liver tissue indicated that hepatocyte apoptosis gradually increased over the initial 12 h period, but decreased thereafter (**Figures 1D, E**). Moreover, results of this assay also showed a similar trend for serum ALT and AST, which increased immediately after reperfusion and peaked at 12 h (**Figure 1C**, *P*<0.001 versus sham).

Furthermore, the proportion of ILC2s on CD45+ cells in the liver were analyzed using multicolor flow cytometry. After pre-gating on single and live cells, CD45+cells were gated to exclude non-hematopoietic cells, such as hepatocytes. In this way, lineage-CD90.2+ST2+CD25+ cells could now be delineated as intrahepatic ILC2s (the Gating strategy is presented in **Supplementary Figure 1A**). We found that there was an increase in the ratio of ILC2s among CD45+ cells as hepatic IRI progressed, peaking at 12h post-IRI (**Figures 1F, G** P<0.05). Moreover, the number of ILC2s per gram of liver tissue gradually increased after reperfusion (**Figure 1G**). Based on these findings we propose that ILC2s may be critically involved in the regulation of hepatic IRI.

### M2 CD45+CD11b+F4/80high macrophages vary as a function of IRI progression

**Figure 2:**
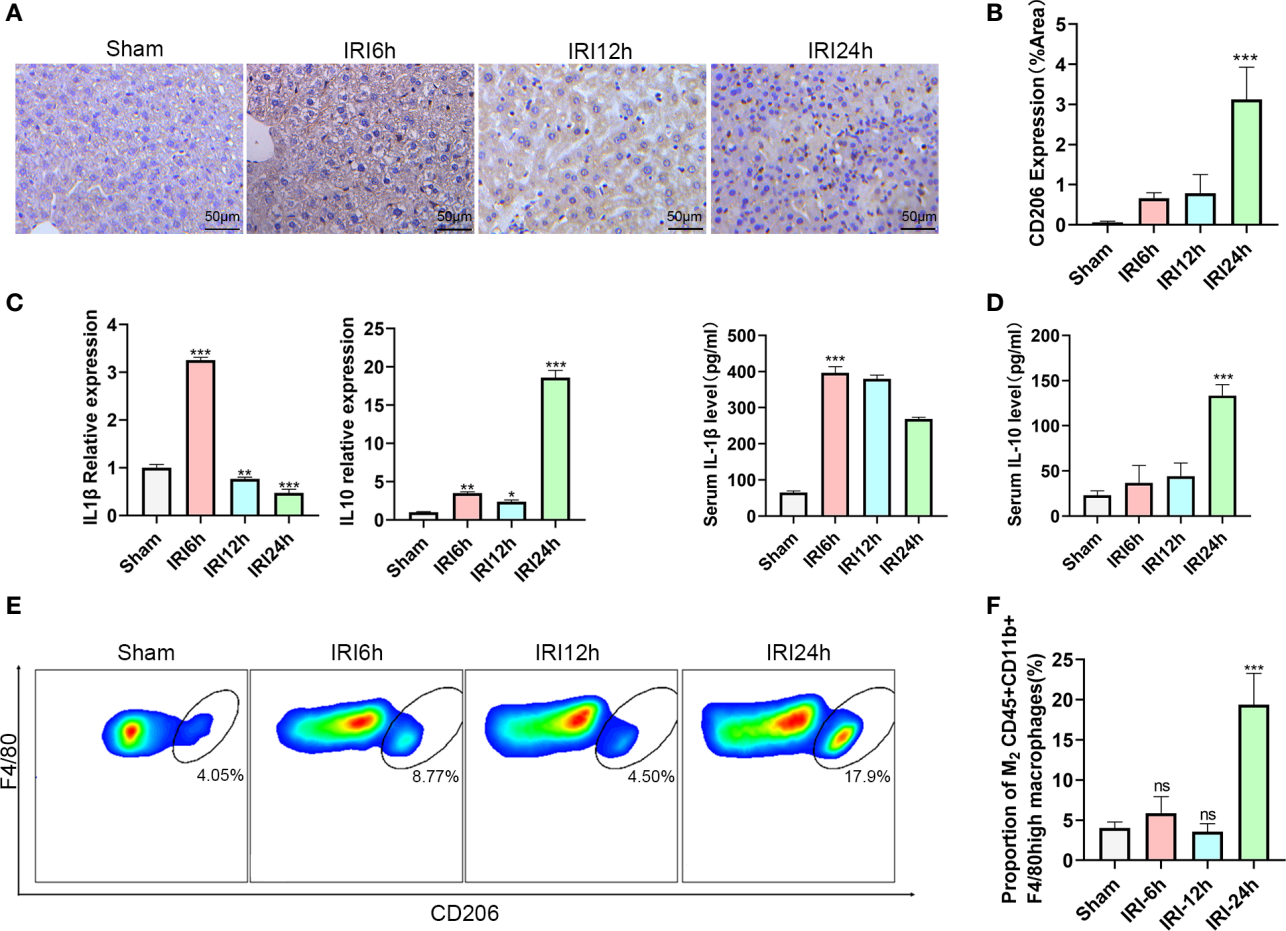
Proportion of M2 CD45+CD11b+F4/80high macrophages varies as a function of hepatic IRI stage. **(A)** Immunohistochemistry assay results demonstrating the changes in CD206 as a function of IRI time. **(B)** Semi-quantification of CD206 expression. **(C)** Relative gene expressions of IL-1β and IL-10 in macrophages purified from mice liver at 6,12 or 24 h post-IRI and sham controls. **(D)** Serum IL-1β and IL-10 levels in Sham and IRI groups at different post-reperfusion time periods. **(E)** Representative FACS analysis showing the percent of M2 CD45+ CD11b+ F4/80high macrophages in sham, IRI6h, IRI12h, IRI24h. (An CD206+ increase in proportion was assumed as a M2 polarization among CD45+CD11b+ F4/80high cells) **(F)** Histograms of the percent of M2 CD45+CD11b+F4/80high macrophages (n = 5 per group). (ns *P*>0.05, *P<0.05,**P<0.01,***P<0.001 versus sham).

During hepatic IRI, we not only observed changes in levels of liver intrahepatic ILC2s, but also in CD45+CD11b+F4/80high macrophages, especially in proportions of the M2 type. To further evaluate these findings, we first determined expressions of CD206 in response to hepatic IRI using immunohistochemistry. CD206 expression gradually increased, showing a slight increase at 12 h after reperfusion and then a significant increase at 24 h (**Figures 2A, B**). Results from flow cytometry analysis revealed that a similar trend was observed for M2 CD45+CD11b+F4/80high CD206+ macrophage ratios (increases in the proportion of CD206+ cells were assumed to represent a M2 polarization among CD45+CD11b+ F4/80high cells), increasing slightly at 6 h, then decreasing at 12 h and finally reaching maximal levels at 24 h (**Figures 2E, F** *P*<0.001 versus sham). Moreover, we also detected mRNA and serum protein levels of IL-1β and IL-10 in these macrophages, with IL-1β being significantly increased at 6 h after IRI, then gradually decreasing to minimal levels at 24 h. In contrast, the M2 CD45+CD11b+F4/80high macrophage related cytokine, IL-10, showed a slight increase at 6 h, followed by a marked rise at 24 h (**Figures 2C, D**; *P*<0.001 versus sham). Chronologically, M2 CD45+CD11b+F4/80high macrophage ratios increased after that of ILC2s, leading us to speculate that the polarization of M2 may be promoted by the increases in ILC2s.

### ILC2s alleviate hepatic IRI by increasing the proportion of M2 CD45+CD11b+F4/80high macrophages

**Figure 3:**
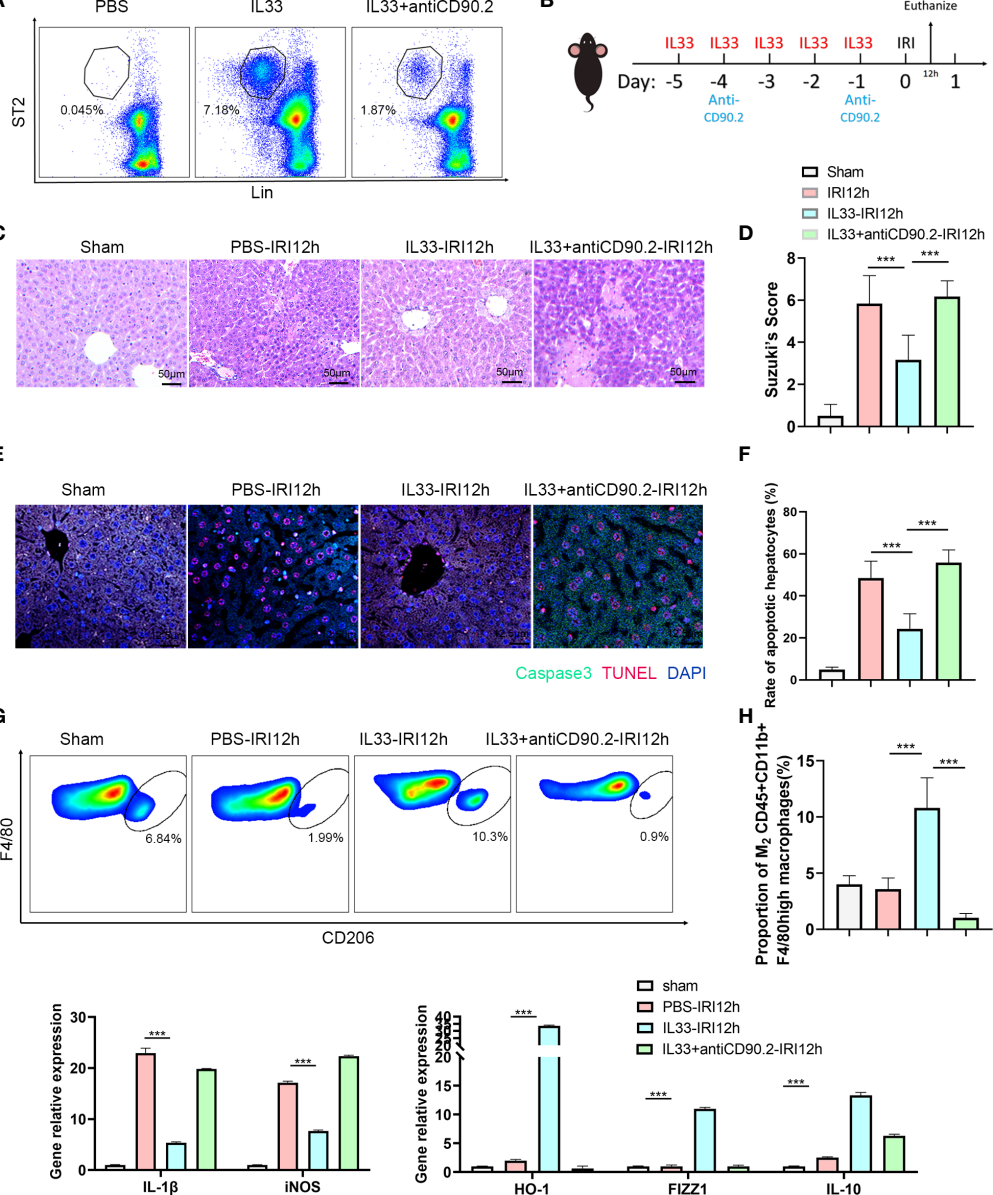
ILC2s protect liver from IRI by increasing the proportion of M2 CD45+CD11b+F4/80high macrophages **(A)** Representative FACS analysis showing that exogenous IL-33 administration significantly increased the proportion of ILC2s in liver as compared with PBS controls and IL-33 receiving anti-CD90.2 administration, which significantly depleted ILC2s. **(B)** Sketch map of medicine administration and IRI surgery. Mice were treated with exogenous IL-33 daily for 5 consecutive days as well as anti-CD90.2 antibody twice before IRI surgery. Mice were euthanized 12 h after reperfusion. **(C)** HE staining of Sham, PBS, IL-33 and IL33&anti-CD90.2 groups as determined at 12 h after reperfusion (×200). **(D)** Suzuki’s Scores resulting from IR-induced liver injury in Sham, PBS, IL-33 and IL33&anti-CD90.2 groups (n=6 per group). **(E)** Representative cell apoptosis immunofluorescence of the 4 groups as determined at 12 h post-reperfusion. Cell apoptosis was measured using TUNEL (red) and Caspase3 (green). Apoptotic cells display a red nucleus (TUNEL) and green cytoplasm (Caspase-3) while normal cells show blue nuclei (DAPI) (×400). **(F)** Histograms of quantitative analysis of TUNEL-positive cells (n = 6 per group). **(G)** Representative FACS analysis showing the proportion change of M2 CD45+CD11b+F4/80high macrophages. (An CD206+ increase in proportion was assumed as a M2 polarization among CD45+CD11b+ F4/80high cells) **(H)** Quantitative analysis of percent of M2 CD45+CD11b+F4/80high macrophages (n = 5 per group). **(I)** Relative gene expressions of IL-1β, iNOS, HO-1, FIZZ1 and IL-10 in macrophages purified from mice liver in different groups. (***P<0.05, ***P<0.001).

To determine whether ILC2s could alleviate hepatic IRI, we performed manipulations in which ILC2s levels were either increased or decreased in a mouse model (Recombinant mouse IL-33 for 5 consecutive days before IRI to stimulate ILC2s and anti-CD90.2 on day -4 and -1 before IRI to deplete ILC2s **Figure 3B**). In mice receiving recombinant mouse IL-33 there was a substantial increase in intrahepatic ILC2s, while these levels were markedly decreased in those receiving IL33+anti-CD90.2 (**Figure 3A**). HE staining, as performed in the harvested liver of these IRI mice at 12 h after reperfusion, revealed little, if any, observation of liver necrosis in mice injected with IL-33, while a considerable amount of necrosis was observed in the PBS and IL33+antiCD90.2 group. However, mild hepatocyte edema and small vessel congestion were present in sections from the IL-33 injected group as compared with sham mice (**Figure 3C**). Results from Suzuki’s Scores indicated that liver injuries within the IL-33 group were less than that observed in the PBS group after reperfusion and, there were more serious damage in IL33+antiCD90.2 groups than IL-33 Group (**Figure 3D**), suggesting that ILC2s had a protective effect on hepatic IRI. In order to further verify the protective effect of ILC2s upon hepatic IRI, the extent of hepatocyte apoptosis was determined in these mice at 12 h after reperfusion. As shown in **Figure 3E**, results from our immunofluorescent assay revealed that a large proportion of hepatocyte nuclei were stained red (TUNEL) and the cytoplasm green (Caspase-3) in mice injected with PBS or IL33+antiCD90.2, indicating that a substantial amount of hepatocyte apoptosis was present. In contrast, the percent of TUNEL and Caspase-3 positive cells were significantly decreased in the IL-33 injection group (**Figure 3F**; *P*<0.001). We also found that following ILC2s proliferation, as achieved with an exogenous injection of IL-33, a marked increase in M2 CD45+CD11b+F4/80high macrophages was observed in these mice when compared with that in the PBS group, as based on FACS analysis (**Figures 3G, H**). In order to determine the relative proportion of M1 versus M2 CD45+CD11b+F4/80high macrophages, these were isolated from liver tissue and their cytokine contents were assessed. As shown in **Figure 3I**, the pro-inflammatory factor IL-1β and iNOS decreased significantly in the IL-33 treated group, while the anti-inflammatory factors HO-1, FIZZ1 and IL-10 were significantly increased. Such results suggest that exogenous IL-33 modulates CD45+CD11b+F4/80high macrophage polarization *in vivo*. However, with ILC2s depletion, as achieved with the administration of anti-CD90.2, these alterations in CD45+CD11b+F4/80high macrophages were substantially attenuated. Therefore, we hypothesized that the protective effect of ILC2s in IRI may, in part, result from the promotion of M2 polarization of CD45+CD11b+F4/80high macrophages.

### Macrophage depletion attenuates the protective effects of exogenous IL-33 in hepatic IRI

**Figure 4:**
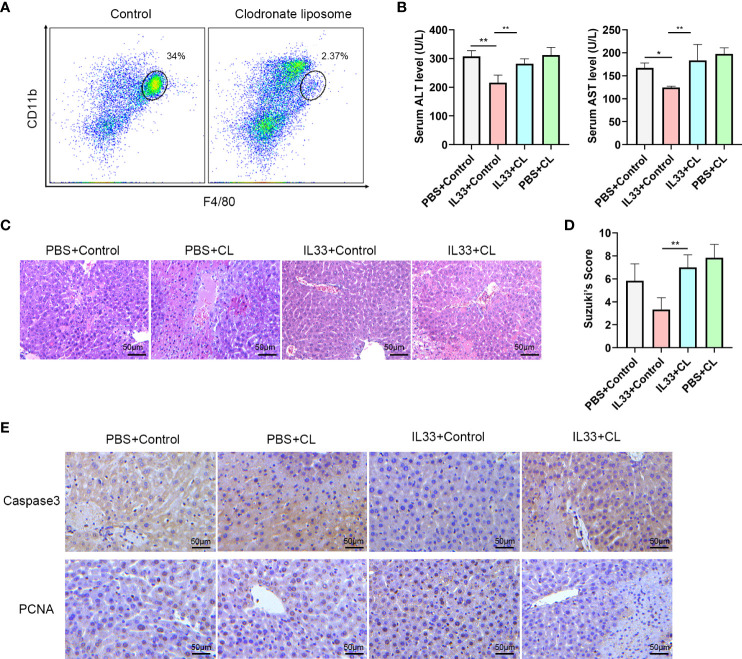
Macrophages depletion prevents protective effects of exogenous IL-33 on hepatic IRI **(A)** Representative FACS analysis showing depletion of macrophages following administration of Clodronate Liposomes (CL). **(B)** Serum ALT and AST levels of mice in PBS+control, PBS+CL, IL33+control and IL33+CL groups 12 h after IRI. **(C)** Histopathological changes in livers within the different treatment groups 12 h after IRI. **(D)** Suzuki’s Scores quantifying the capacity for CL to attenuate the protective effects of exogenous IL-33 against hepatic IRI (n=6 per group) (mice were Euthanized 12h after IRI). **(E)** Histochemistry of caspase3 and PCNA in the four groups (mice were euthanized 12h after IRI). (*P<0.05, **P<0.01).

In order to determine whether CD45+CD11b+F4/80high macrophages are essential for the protective effect of exogenous IL-33 in hepatic IRI, we assessed the effects of macrophages depletion in this mouse model. This was achieved with an administration of Clodronate Liposomes (CL). As shown in **Figure 4A**, there was a significant reduction in CD45+CD11b+F4/80high macrophages in response to this CL treatment. We euthanized mice 12 hours after hepatic IRI to detect hepatic structural and functional injury. With this depletion of macrophages, the protective effects of exogenous IL-33 on hepatic IRI were no longer present and serum ALT and AST levels of mice treated with IL-33+CL were significantly greater than that in mice treated with IL-33+Control (**Figure 4B**). Results from our histological analysis corroborated these findings, as mice injected with IL-33 showed significantly less necrosis as compared with that of the other groups, while severe necrosis, hepatocyte edema and vessels congestion were observed in mice treated with IL-33+CL (**Figure 4C**). Suzuki’s Scores of the IL33+CL group were significantly greater than that of the IL33+Control group, indicating that macrophages depletion weakened the protection of exogenous IL-33 against hepatic IRI (**Figure 4D**). Similarly, when assessing apoptosis, we found that IL-33 treatment significantly reduced the proportion of Caspase-3 positive cells while remarkably increasing PCNA positive cells, indicating that IL-33 administration reduced liver apoptosis and promoted liver regeneration in hepatic IRI. When compared with that of the IL-33 group, the number of Caspase-3 positive cells in the IL-33+CL group was significantly increased while PCNA positive cells remarkably decreased, implying that the depletion of macrophages severely attenuated the ability for ILC2s to resist apoptosis and promote regeneration in hepatic IRI (**Figure 4E**).

### IL-4 polarizes CD45+CD11b+F4/80high macrophages to the M2 type *via* the JNK/Stat3 pathway

**Figure 5:**
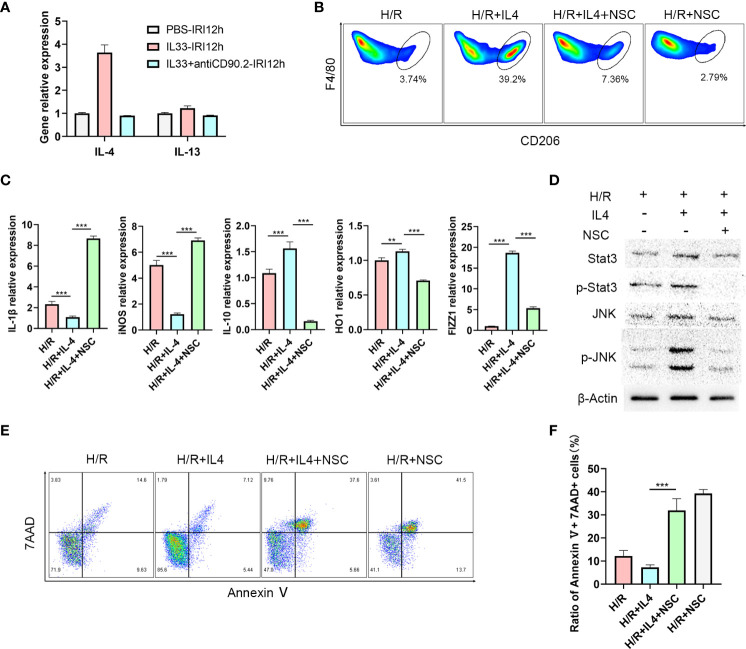
IL-4 polarizes CD45+CD11b+F4/80high macrophages to M2 type *via* the JNK/Stat3 pathway **(A)** Relative expression levels of IL-4 and IL13 mRNA in liver tissue of mice treated with IL-33 and IL-33+antiCD90.2 at 12 h following IRI. **(B)** Representative FACS analysis showing M2 CD45+CD11b+F4/80high macrophages proportion changes of treated with IL-4 or IL-4+Stat3 inhibitor NSC74859 (abbreviated as NSC) at 12 h following hypoxia and reoxygenation. (An increase CD206+ in proportion was assumed as a M2 polarization among CD45+CD11b+ F4/80high cells) **(C)** IL1β, iNOS, HO-1, IL-10 and FIZZ-1 mRNA expression levels in macrophages within the different groups were determined using quantitative PCR. **(D)** Stat3, p-Stat3, JNK and p-JNK protein levels were assessed using western blot in RAW264.7 cells at 12 h after hypoxia and reoxygenation in the presence or absence of IL-4 or Stat3 inhibitor. **(E)** Apoptosis of AML12 cells after co-culture with different macrophages. **(F)** Histograms of quantitative analysis of 7AAD and Annexin V double positive cells. (**P<0.01, ***P<0.001).

ILC2s mainly exert their effects by secreting Th2 cytokines such as, IL-4, IL-5 and IL-13 (24). To determine which cytokine may be critical for the M2 phenotype transformation mediated by ILC2s, we examined mRNA levels of these cytokines within the liver. As expected, IL-4 and IL-13 expression levels in liver tissue increased after IL-33 injections, and IL-4 was clearly greater than that of IL-13 (**Figure 5A**). Moreover, in IL-33+antiCD90.2 group, the increase of IL-4 decreased significantly. Therefore, we considered that IL-4 may serve as the cytokine through which ILC2 regulates phenotypic changes in CD45+CD11b+F4/80high macrophages. To test this hypothesis, primary macrophages were extracted from C57BL/6 mice and treated with IL-4 or an IL-4+Stat3 inhibitor NSC74859 (abbreviated as NSC). When IL-4 was added to the culture medium for 24 h prior to hypoxia and reoxygenation (H/R), the proportion of M2 macrophages was significantly increased. However, this trend for M2 macrophage polarization was clearly reduced when NSC was combined with IL-4 (**Figure 5B**). In addition, cells treated with IL-4 had reduced expressions of M1 macrophage markers, including IL-1β, iNOS, and enhanced expressions of M2 macrophage markers, including IL-10, FIZZ1 and HO-1 (**Figure 5C**). From the level of protein assessment, as performed using western blot, IL-4 administration was shown to upregulate the expressions of p-Stat3 and p-JNK, while treatment with the IL4+Stat3 inhibitor downregulated these expressions (**Figure 5D**). Finally, we co-cultured hepatocyte AML12 cells with macrophages treated with IL4 or IL4+Stat3 inhibitor and then analyzed the degree of apoptosis within these AML12 cells using flow cytometry analysis. As shown in **Figure 5E**, after 1 h of hypoxia and 12 h of reoxygenation, AML12 cells showed a considerable degree of apoptosis, while those co-cultured with IL4-treated macrophages demonstrated a significant reduction in hepatocyte apoptosis. However, when co-cultured with macrophages treated with IL-4+NSC, the apoptosis within these hepatocytes was significantly increased (P<0.001) (**Figure 5F**).

Taken together, the findings are consistent with the hypothesis that ILC2s alleviate hepatic IRI by promoting M2 polarization of CD45+CD11b+F4/80high macrophages through the IL-4/JNK/Stat3 pathway. Whether other cells are involved in the release of IL-4 awaits further research. Such findings provide the foundation for the development of novel strategies to alleviate hepatic IRI as achieved through modulation of ILC2.

## Discussion

Hepatic IRI, represents a significant risk factor affecting the prognosis of hepatectomy or liver transplantation (25). The mechanisms of hepatic IRI are complex, involving oxidative stress, apoptosis, autophagy, pyroptosis and immune factors (26, 27), and it is also certain that resident liver macrophages, play an important role in this liver injury process (28–30). According to the different functions exerted by macrophages, they can be divided into M1 macrophages that promote or M2 macrophages that inhibit inflammation (31). A number of studies have focused on procedures involved with increasing the proportion of M2 macrophages as an approach to reduce IRI (6, 32, 33). Our current results show that with an exogenous administration of IL-33, ILC2s are increased along with a proportional increase in M2 CD45+CD11b+F4/80high macrophages, effects which are accompanied with a reduction in IRI. Such effects can be attenuated following ILC2s depletion, as achieved with anti-CD90.2. Accordingly, it appears that ILC2s proliferation can alleviate IRI through the promotion of M2 macrophage polarization

While the results of our present study provide the first evidence for a role of ILC2s in hepatic IRI, the role of exogenous IL-33 in hepatic IRI has been described previously by Li and his colleagues (20). They demonstrated that the effects of exogenous IL-33 in hepatic IRI were associated with a Th1 to Th2 cytokine type shift and, in this way, exogenous IL-33 may suppress inflammation of hepatic IRI by inducing IL-4, IL-5 and IL-13 release. Interestingly, ILC2s represent a heterogeneous group of immune cells which can be activated by IL-33 and IL-25 and mainly mediate type 2 inflammation by releasing IL-4, IL-5 and IL-13 (34–36). Such a cascade of events most likely serves as a medium for IL-33 to play its role in hepatic IRI.

Endogenous IL-33 is immediately released during liver IRI and contributes to early tissue injury as an alarmin (23). Discrepant effects of exogenous IL-33 on hepatic IRI have been reported. Yazdani demonstrated that exogenous IL-33 exacerbates liver IRI by amplifying a neutrophil extracellular trap formation causing excessive sterile inflammation (22), However, it has also been reported that exogenous IL-33 significantly reduces hepatocellular injury in IRI and liver neutrophil accumulation (21). Such variations in results may be attributable to differences in doses and/or the timing of exogenous IL-33 administration. Yazdani used a high dose (up to 10 ug) which was administered at 1 h before or immediately after ischemia, while in our study a low dose, long-course pretreatment of IL-33, consisting of a daily dose of 0.3 ug for 5 consecutive days, was administered prior to IRI surgery. With our protocol, a milder effect was achieved, enabling this pretreatment sufficient time for the immune system to alter the proportion and phenotype of ST2-expressing cells, particularly ILC2s, to prepare for the future injury. It is also possible that this exogenous IL-33 pretreatment may alter other cells expressing ST2 *in vivo*, such as, regulatory T (Treg) cells, TH1 cells, CD8+ T cells, natural killer (NK) cells (37) and iNKT cells (38). Notably, Ngo reported that an exogenous injection of IL-33 induced a remarkable increase of Th2 associated cytokines accompanied with an accumulation of Tregs and ILC2s in the colon, which could play a protective role in severe acute colitis (39). Our findings suggest a specific role for ILC2s, as depletion with the anti-CD90.2 antibody substantially reduced the protective effect of exogenous IL-33 on IRI. Such results strongly suggest that the IL-33 induction of ILC2s was the critical component for this protective role in hepatic IRI.

It appears that the *in vivo* regulation of liver resident macrophage polarization by IL-33 is critically dependent on ILC2s. Previously, we have directly treated liver resident macrophages with recombinant mouse IL-33 *in vitro* and failed to observe any of the production changes in inflammatory factors which could be observed when recombinant mouse IL-33 is administered *in vivo*. Similar results were reported by Li and Sakai et al (20, 21), who found that ST2, the receptor for IL-33, was not expressed by liver resident macrophages, thereby demonstrating that endogenous IL-33 exerts a hepatoprotective effect as a result of increased NF-κB and Bcl-2 expression in hepatocyte and is independent of KCs. However, results from several studies have indicated that IL-33/ILC2s regulate M2 polarization of macrophages (40–42) and that IL-33 can stimulate ILC2 proliferation (19, 43, 44), leading to the conclusion that although IL-33 is capable of regulating the polarization of macrophages *in vivo*, this ability is reliant on ILC2s. Our findings, as described above, suggest that the protective effects of ILC2s in hepatic IRI may rely on exogenous IL-33. In the ILC2s depletion group, the pathological damage observed within liver tissue was essentially the same as that of the control group (**Supplementary Figure 1B**) and Suzuki’s Score between the two groups were not significantly different (**Supplementary Figure 1C**). In this way, in the absence of exogenous IL-33, ILC2s may be redundant in the hepatic ischemia-reperfusion injury. Such a phenomenon has been observed in renal IRI (45).

In our hepatic IRI model, IL-4 increased significantly at 12 h after reperfusion, which represents an important stimulatory component for shifting of the macrophage phenotype to the M2 type. However, the major sources for IL-4 remain unclear. In addition to ILC2s (46), there exist several types of cells capable of producing IL-4, such as CD4+Th cells, Treg cells (39) and iNKT (38). Any of these cells may provide a source for IL-4 in the liver IRI model. Instead of individually screening IL-4 producing candidate cells, we eliminated the effects of ILC2s with use of a monoclonal antibody. The reduction in IL-4 after CD90.2 administration suggests that ILC2 could produce the IL-4 necessary for promoting this change to the M2 phenotype of macrophages. However, it should be noted that ILC2s are not the only source of IL-4, as iNKT cell-derived IL-4 could also be involved in promoting M2-like macrophages during hepatic IRI as reported by Goto et al (47). Accordingly, whether other cells are involved in this release of IL-4 will require further investigation.

Intravenous injection with Clodronate Liposomes depleted macrophages in organs where liposomes had an unhindered access to. When macrophages in the liver are depleted, those in the spleen are also cleared, thereby alleviating the potential for macrophage recruitment to the liver after IRI. However, with an administration of IL-33, the ratio of M2 type macrophages both in the liver and spleen are increased. These “protective” macrophages would then be available for recruitment from the spleen to the liver in response to hepatic IR. In this way, the existence of “protective” macrophages as induced by IL-33 may not only involve the liver but also the spleen as a very likely source.

In conclusion, we have demonstrated that the proliferation of ILC2s, as stimulated by exogenous IL-33, can alleviate hepatic IRI, primarily by inducing the polarization of CD45+CD11b+F4/80high macrophages to the M2 type. The possible molecular mechanisms for this process may, in part, involve the secretion of IL-4 by ILC2s *via* regulation of the JNK/Stat3 pathway in CD45+ CD11b+ F4/80high macrophages. These findings demonstrating the immune mechanisms of ILC2s in the alleviation of hepatic IRI can serve as the foundation for the development of new targets and strategies to prevent the injuries in liver function that accompany liver surgery.

## Materials and methods

### Animals

Male C57BL/6 mice (8-10 weeks old, 23 ± 2g) were purchased from the SiPeiFu (Beijing) Biotechnology Co., LTD [license SYXK (Beijing) 2017-0010] and were housed under specific pathogen-free condition with free access to water and food. The animal welfare and use protocol was approved by the Animal Ethics Committee of the Beijing Friendship Hospital of Capital Medical University.

### Mouse model and treatment

The segmental (70%) hepatic ischemia model was employed in these experiments as described previously (48). Briefly, after a midline abdominal excision, the hepatic pedicle was carefully exposed and arterial and portal venous blood supplies to the cephalad lobes were clamped for 1 h using a nontraumatic sterile clamp. After this ischemic period, the clamps were released to induce reperfusion. Animals were euthanized at 6, 12 or 24 h post-reperfusion. Mice in the sham group were subjected to an identical procedure, except for the vascular occlusion.

For exogenous IL-33 administration, 0.2 ug of mouse recombinant IL-33 (Absin, abs04085) was injected through caudal vein daily for 5 consecutive days prior to the IRI treatment (43). Control animals received PBS only. Macrophages depletion was induced by administration of 10 mg/kg Clodronate Liposomes (LIPOSOMA C-005) *via* caudal vein injection as administered once at 24 h prior to IRI treatment. Anti-CD90.2 antibody (Biolegend, 105352) was injected into teil vein at -4 and -1 day before IRI surgery.

### Intrahepatic lymphocytes and macrophages isolation

Intrahepatic lymphocytes and macrophage extraction was performed as described in previous studies (43, 49). Briefly, intrahepatic lymphocytes were obtained with use of *in vitro* collagenase type IV (Sigma, USA) digestion and discontinuous density gradient centrifugation by Ficoll (GE, 17-5442-02). Macrophages were isolated from the liver by *in situ* collagenase type IV digestion and discontinuous density gradient centrifugation by 25%-50% Percoll. Then, anti-F4/80 MicroBeads UltraPure, (Miltenyi Biotec, Germany, 130-110-443) were used to sort macrophages for further culturing or RNA extraction.

### Histology, immunohistochemistry and immunofluorescence

Formalin fixed and paraffin embedded liver tissue was cut into 4 μm-thick sections and stained with H+E to evaluate the degree of liver damage. Suzuki’s Scores were calculated to quantify the damage. To identify apoptosis and regeneration of hepatocytes resulting from hepatic IRI, Caspase 3 (1:200, ab109201; Abcam), PCNA (1:200, ab92552; Abcam) and CD206 (1:200, ab252921; Abcam) rabbit antibodies were used for immunohistochemical staining. Image J was used to semi-quantitative analysis. Terminal deoxynucleotidyl transferase dUTP nick-end labeling (TUNEL) reaction was performed using an *In Situ* Cell Death Detection Kit, TMR red to assess apoptosis. The mean number of TUNEL-positive cells in five different fields (400×) were averaged for quantification.

### Serology detection

Serum ALT, AST and cytokine levels of IL-4, and IL1β were assayed by ELISA according to instructions of the manufacturer (all ELISA kits were purchased from R&D Systems). All samples were measured in duplicate.

### Cell culture and treatments

RAW264.7 and AML12 cell lines were purchased from the Procell Life Science and Technology Co., Ltd. (cat no.: CL-0190; Wuhan, China). Primary macrophages extracted from C57BL/6 mice were cultured with DMEM medium containing 10% FBS and 1% Penicillin+ Streptomycin mixture. AML12 cell lines were cultured with DMEM/F12 medium containing the same concentration of FBS and antibiotics as described above.

RAW 264.7 cells were plated in 6-well plates at a density of 4×10^5^ cells/well and were divided into 3 groups: 1) Hypoxia and reoxygenation (H/R) - cells were immersed in paraffin oil for 1 h to produce an anoxic environment and then cultured in DMEM or DMEM/F12 for 12 h to induce hypoxia and reoxygenation injuries, 2) H/R+IL-4 - cells were treated with 20 ng/ml mouse recombinant IL-4 (CST, 5208SC) for 24 h before the H/R procedure and 3) H/R+IL-4+Stat3 inhibitor - cells were treated with 100 μM NSC 74859 (S3I-201) (MedChemExpress, HY-15146.) for 24 h following the H/R procedure. AML12 were co-cultured with RAW264.7 cells from the different groups during hypoxia and reoxygenation and then remained in these co-cultures for 12 h.

### Flow cytometry analysis

ILC2s were defined as lineage negative (Lineage: TCRβ, TCRγ/δ, CD4, CD11c, CD5, CD8a, NK1.1, CD11b, ly-6G (Gr-1), TER-119, CD45R (B220), CD3e) and CD45, CD90.2 (Thy1), ST2, CD25 positive cells. Macrophages were suspended as single cells and stained with antibodies to CD45 (30-F11) Rat mAb (FITC Conjugate), CD11b/ITGAM (M1/70) Rat mAb (PE Conjugate), F4/80(BM8) Rat mAb (AF700 Conjugate) or CD206(C068C2) Rat mAb (BV711 Conjugate). (**Supplementary Table 1**) All antibodies were purchased from CST or BioLegend. Cells were analyzed on an Attune NxT flow cytometer (Thermo Fisher). CD206 was regarded as a marker for M2 macrophages. FlowJo software (Tree Star Inc., Ashland, OR, USA) was used to analyze the results. Annexin V (FITC Conjugated) and 7AAD (PE Conjugated) were used to test apoptosis conditions.

### QRT-PCR

Total RNA was extract from liver tissue or RAW264.7 cells or purified macrophagess using TRIzol (absin, abs60154) according to operation steps of the instructions. Total RNA (1500ng) was then reversed with use of a TRUEscript 1st Strand cDNA Synthesis Kit (PC1802; Aidlab). Real-time PCR assays were performed on the ABI7500 Fast system (Applied Biosystems) using SYBR mastermix (Invitrogen) according to manufacturers’ instructions.

### Western blotting

Protein samples from liver tissue and RAW264.7 cells were extracted using RIPA buffer with a protease inhibitor (P0013B; Beyotime, Shanghai, China). Protein samples were separated *via* 8–12% SDS-PAGE (Bio-Rad, Redmond, WA, USA), electroblotted onto polyvinylidene difluoride membranes (Billerica, MA, USA), and then incubated with primary anti-Caspase-3 (1:2000, ab184787; Abcam), anti-BAX (1:2000, ab182733; Abcam), anti-Bcl2 (1:2000, ab182858; Abcam), anti-Stat3 (1:2000, #30835; CST), anti- phosph-JNK (1:2000, ab239886; Abcam) or anti-β-actin (1:2000, #4970; CST) rabbit antibodies at 4°C overnight. Horseradish-peroxidase-conjugated anti-rabbit IgG was used as the secondary antibody (1:3000; Cell Signaling Technology). Antibody binding was detected using a chemiluminescence system (Tanon-5200 Multi; Shanghai, China). Image J was used to semi-quantitative analysis.

### Statistical analysis

Data are shown as Means ± SEMs. Differences among groups (≥3) were performed by one-way ANOVA with the *post-hoc* Tukey test used for pairwise comparisons of subgroups. A student’s t-test was used to compare data between two groups. The SPSS 19.0 was used for conducting these analyses. P < 0.05 was required for results to be considered as statistically significant.

## Data availability statement

The original contributions presented in the study are included in the article/**supplementary material**. Further inquiries can be directed to the corresponding author/s.

## Ethics statement

The animal study was reviewed and approved by Animal Ethics Committee of Beijing Friendship Hospital, Capital Medical University.

## Author contributions

Z-JZ, L-YS, X-JC and H-MZ participated in the research design. S-PL, J-MZ and L-XZ performed the molecular investigations; S-PL and X-JC participated in *in vivo* and *in vitro* experiment. JS, BC and G-PZ performed the data management and statistical analyses after discussion with all authors. S-Pi and X-JC wrote the manuscript. All authors contributed to the article and approved the submitted version.

## Funding

This work was supported by the National Natural Science Foundation of China (No. 81970562).

## Conflict of interest

The authors declare that the research was conducted in the absence of any commercial or financial relationships that could be construed as a potential conflict of interest.

## Publisher’s note

All claims expressed in this article are solely those of the authors and do not necessarily represent those of their affiliated organizations, or those of the publisher, the editors and the reviewers. Any product that may be evaluated in this article, or claim that may be made by its manufacturer, is not guaranteed or endorsed by the publisher.
