# Supplementary figures and images for: ILC2s expanded by exogenous IL-33 regulate CD45+CD11b+F4/80high macrophage polarization to alleviate hepatic ischemia-reperfusion injury
Source: Front Immunol. 2022 Jul 29;13:869365. doi: 10.3389/fimmu.2022.869365 (PMC9372719; doi:10.3389/fimmu.2022.869365)

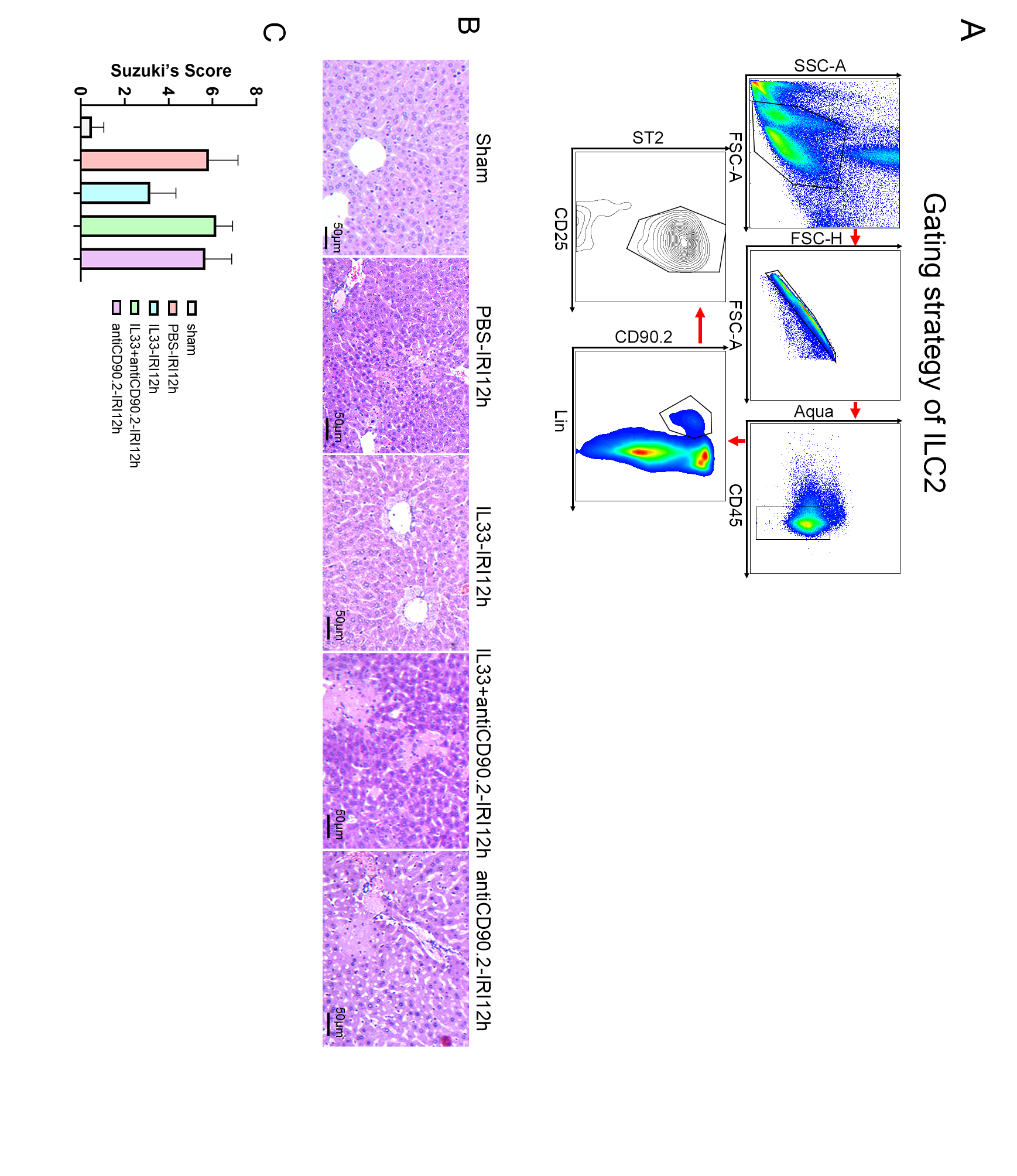

Supplement: Supplementary file 1 [file Image_1.tif]
