# Supplementary material for: ILC2s expanded by exogenous IL-33 regulate CD45+CD11b+F4/80high macrophage polarization to alleviate hepatic ischemia-reperfusion injury
Source: Front Immunol. 2022 Jul 29;13:869365. doi: 10.3389/fimmu.2022.869365 (PMC9372719; doi:10.3389/fimmu.2022.869365)
Supplement: Supplementary file 2 [file Table_1.docx]

**Flow cytometry antibody list**

**ILC2**

| **Antibody** | **Clone** | **Fluor** | **Catalog** | **Company** |
| --- | --- | --- | --- | --- |
| **Mouse** **Hematopoietic Lineage Biotin Panel** | — | Biotin | 88-7774-75 | eBioscience |
| **TCRβ** | H57-597 | Biotin | 109203 | BioLegend |
| **TCRγ/δ** | GL3 | Biotin | 118103 | BioLegend |
| **CD4** | GK1.5 | Biotin | 100404 | BioLegend |
| **CD11c** | N418 | Biotin | 117303 | BioLegend |
| **CD5** | 53-7.3 | Biotin | 100603 | BioLegend |
| **CD8a** | 53-6.7 | Biotin | 100704 | BioLegend |
| **NK1.1** | PK136 | Biotin | 108703 | BioLegend |
| **Streptavidin** | — | FITC | 11-4317-87 | eBioscience |
| **LIVE/DEAD™ Fixable Aqua Dead Cell Stain Kit** | — | 405 nm excitation | L34965 | Invitrogen |
| **CD45** | 30-F11 | APC-Cy7 | 103116 | BioLegend |
| **CD25** | PC61.5 | PE-Cy7 | MA5-17815/25-0251-81 | eBioscience |
| **CD90.2** | 53-2.1 | PE-Cy7/eFluor450 | 25-0902-81 | eBioscience |
| **CD127** | A7R34 | Brilliant Violet 421 | 135027 | BioLegend |
| **IL33R/ST2** | DIH9 | PE/APC | 145304 | BioLegend |

**PS：**

**Mouse Hematopoietic Lineage Biotin Panel**

following antibodies:

1. Biotin anti-mouse CD3 (145-2C11): 0.25 μg/2 μL (0.125 mg/mL)

2. Biotin anti-mouse CD45R/B220 (RA3-6B2): 0.06 μg/2 μL (0.03 mg/mL)

3. Biotin anti-mouse CD11b (M1/70): 0.06 μg/2 μL (0.03 mg/mL)

4. Biotin anti-mouse Erythroid marker (TER-119): 0.06 μg/2 μL (0.03 mg/mL)

5. Biotin anti-mouse Ly-6G (RB6-8C5): 0.03 μg/2 μL (0.015 mg/mL)

**Kupffer**

| **Antibody** | **Clone** | **Fluor** | **Catalog** | **Company** |
| --- | --- | --- | --- | --- |
| **F4/80** | BM8 | AF700 | 123130 | BioLegend |
| **CD206** | C068C2 | BV711 | 141727 | BioLegend |
| **CD11b** | M1/70 | PE | 24965S | CST |
| **LIVE/DEAD™ Fixable Aqua Dead Cell Stain Kit** | — | 405 nm excitation | L34965 | Invitrogen |
| **CD45** | 30-F11 | FITC | 62307S | CST |
